# Molecular Modeling of Mechanosensory Ion Channel Structural and Functional Features

**DOI:** 10.1371/journal.pone.0012814

**Published:** 2010-09-16

**Authors:** Renate Gessmann, Nikos Kourtis, Kyriacos Petratos, Nektarios Tavernarakis

**Affiliations:** Institute of Molecular Biology and Biotechnology, Foundation for Research and Technology, Heraklion, Crete, Greece; Duke University, United States of America

## Abstract

The DEG/ENaC (Degenerin/Epithelial Sodium Channel) protein family comprises related ion channel subunits from all metazoans, including humans. Members of this protein family play roles in several important biological processes such as transduction of mechanical stimuli, sodium re-absorption and blood pressure regulation. Several blocks of amino acid sequence are conserved in DEG/ENaC proteins, but structure/function relations in this channel class are poorly understood. Given the considerable experimental limitations associated with the crystallization of integral membrane proteins, knowledge-based modeling is often the only route towards obtaining reliable structural information. To gain insight into the structural characteristics of DEG/ENaC ion channels, we derived three-dimensional models of MEC-4 and UNC-8, based on the available crystal structures of ASIC1 (Acid Sensing Ion Channel 1). MEC-4 and UNC-8 are two DEG/ENaC family members involved in mechanosensation and proprioception respectively, in the nematode *Caenorhabditis elegans*. We used these models to examine the structural effects of specific mutations that alter channel function *in vivo*. The trimeric MEC-4 model provides insight into the mechanism by which gain-of-function mutations cause structural alterations that result in increased channel permeability, which trigger cell degeneration. Our analysis provides an introductory framework to further investigate the multimeric organization of the DEG/ENaC ion channel complex.

## Introduction

The DEG/ENaC family of ion channels is a large group of proteins sharing a high degree of sequence and overall primary structure similarity. Members of the DEG/ENaC family have been identified in organisms ranging from nematodes, snails, flies, vertebrates, including humans, and are expressed in tissues as diverse as kidney epithelia, muscle and neurons [1], [2]. While DEG/ENaC proteins are involved in many diverse biological functions in different organisms, they display strong sequence conservation across species. The extensive sequence similarity indicates that DEG/ENaC family members shared a common ancestor relatively early in evolution and a highly conserved overall structure [3].

DEG/ENaC proteins range from about 550 to 950 amino acids in length and share several distinguishing blocks of sequence similarity. Subunit topology is invariable: all DEG/ENaC family members have two transmembrane domains, with cysteine-rich domains (CRDs) situated between these two transmembrane segments [2], [4]. DEG/ENaCs are situated in the membrane such that amino- and carboxy-termini project into the cytoplasm while most of the protein, including the CRDs, is extracellular. Highly conserved regions include the two transmembrane helices (TM I and II), a short amino acid stretch before the first transmembrane helix, the extracellular cysteine-rich domains (CRDs), an extracellular regulatory domain and a neurotoxin-related domain (NTD) before the predicted transmembrane helix II. The high degree of conservation of cysteine residues in these extracellular domains suggests that the tertiary structure of this region is critical for the function of most channel subunits and may mediate interactions with extracellular structures. The first of the two transmembrane helices (TM I) is generally hydrophobic, whereas the more carboxy-terminal of these (TM II) is amphipathic. TM II exhibits strong conservation of residues (consensus GLWxGxSxxTxxE) and has been implicated in pore function [5].

All living organisms have developed highly specialized structures that are receptive to mechanical forces originating either from the surrounding environment or from within the organism itself. The mechanisms underlying the capability of living cells to receive and act in response to mechanical inputs emerged early during evolution. Genetic, molecular and electrophysiological studies have implicated specific DEG/ENaC ion channels in mechanotransduction in nematodes, flies and mammals [5]. Therefore, these proteins are strong candidates for a metazoan mechanosensitive ion channel [2]. The *Caenorhabditis elegans* degenerins MEC-4 (*Mec*hanosensory) and UNC-8 (*Unc*oordinated) are two DEG/ENaC family members required for the mechanosensitive behaviors of touch sensation and proprioception, respectively, of the nematode [5]. MEC-4 and the related MEC-10 ion channel subunit form the core mechanosensory ion channel in touch receptor neurons of the animal [6]. UNC-8 likely co-assembles with the related DEG/ENaC protein DEL-1 (Degenerin-like) into a stretch sensitive channel in motor neuron process modulates coordinated movement in response to body stretch [7]. However, the mechanism by which mechanical forces impinge upon mechanosensitive channel subunits to modulate open channel probability remains elusive. Information on the structure and overall organization of the mechanosensitive ion channel is required to address this question.

Recently, the structure of the related chicken ASIC1 ion channel was determined by crystallography [8], [9]. ASIC1 belongs to the family of acid-sensing ion channels (ASICs), which are similar in sequence and are therefore expected to exhibit similar overall topology to the DEG/ENaC ion channels [10]. Members of the mammalian ASIC subfamily are gated by protons and have been implicated in neurotransmission, in the central nervous system [11], [12].

Here, we describe homology-based models of MEC-4 and UNC-8 (accessible at http://elegans.imbb.forth.gr/models/). Four models of MEC-4 and four models UNC-8 subunits were obtained based on the 3 subunit structures of the homo-trimeric, closed acid sensing ion channel (ASIC1) of chicken (PDB ID: 2QTS; [9]) and on the one subunit structure of the minimal function construct of the same channel and organism (PDB ID: 3HGC; [8]). The truncated protein variant used to obtain the first crystal structure (henceforth called 2QTS) comprises 438 amino acids (26–463). Only residues 42–457 (subunit A), 42–461 (subunit B) and 40–457 (subunit C) could be located in the electron density maps at 1.9 Å resolution. This truncated protein variant does not exhibit proton-dependent gating.

The minimal function DNA construct (mfc) used to obtain the second protein crystal structure (henceforth called 3HGC) encodes 466 amino acids (1–466). In this case, only 406 residues (46–451) could be located in the lower resolution (3 Å) electron density map. Structures comprising all visible residues have been used for modeling the MEC-4 and UNC-8 subunits A, B, C and H. These subunit models can be used to determine the location of amino acid residue alterations encoded by mutant alleles, relative to defined channel structural features.

**Figure 1:**
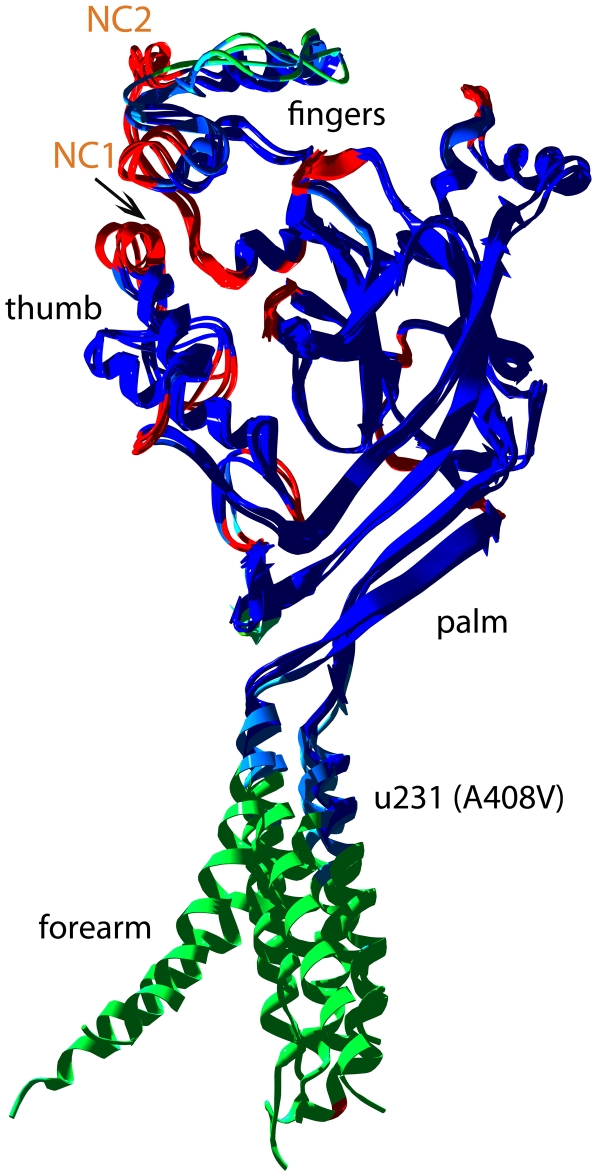
Superposition of the four subunits of MEC-4 modeled on the 3 subunits of the crystal structure of the closed acid sensing ion channel of chicken (PDB ID: 2QTS) and on the minimal function channel (PDB ID: 3HGC). Colors denote the model confidence factor of the SWISS-MODEL server; dark blue regions denote high confidence, green to red gradually lower reliability. Lower reliability is assigned if the template residues (in A, B and C of 2QTS and 3HGC) differ from one another or to sparse loops incorporated from other structures. The arrow marks the position of *e49* in the modeled UNC-8 subunits (not shown). NC1 and NC2 denote the position of two blocks of sequence in MEC-4 which have no counterpart in the alignment and therefore are not modeled.

In addition, based on both structures (3 different subunits forming a trimer in 2QTS and one subunit forming a trimer in 3HGC), 2 models of MEC-4 forming a trimer were derived and used to examine the structural and functional effects of the MEC-4 gain-of-function allele *u231* (A→V). The overall model structures of MEC-4 and UNC-8 resemble an upright forearm and a clenched hand (Figure 1). The forearm is built by two transmembrane helices, which span about 40 Å. The N-terminal and the C-terminal regions of the protein are cytoplasmic and their structure has not been determined by crystallography. The N-terminal region of MEC-4 was previously modeled by homology to the protease procaricain [13]. The hand with the wrist, the palm, the thumb and the fingers constitute the extracellular domains. A β-sheet in the palm spans nearly the entire height (∼55 Å) of the extracellular domain. This β-sheet connects to both transmembrane helices and also to the thumb domain, which is composed of three helices and was suggested to play a role in the transduction of conformational changes in ASIC1 [9].

## Results and Discussion

### Homology-based modeling of MEC-4

**Figure 2:**
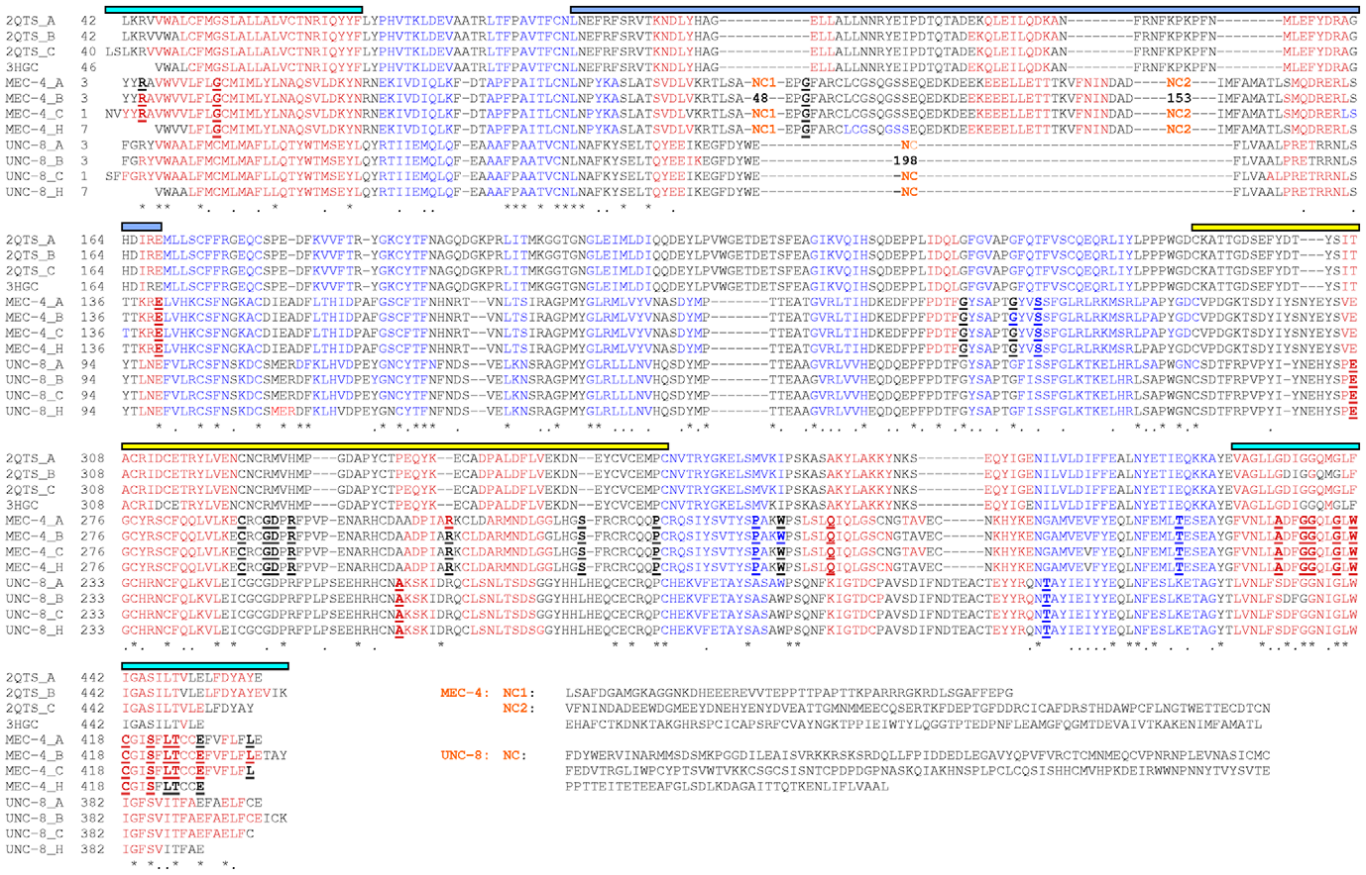
Structural alignment used for modeling of MEC-4 and UNC-8. MEC-4_A, MEC-4_B, MEC-4_C, MEC-4_H and UNC-8_A, UNC-8_B, UNC-8_C, UNC-8_H were treated independently from one another. Each subunit is modeled to the superimposed subunits of 2QTS or 3HGC, respectively. Secondary structural elements are color-coded (red: helices, blue: β-strands). Asterisks below the sequences denote identical, and dots similar residues. Amino acid substitutions corresponding to mutant alleles are depicted in bold and are underlined, for specifics see Table 1. Solid lines above the alignment denote parts of the modeled hand (turquoise: forearm, light blue: fingers, yellow: thumb).

Subunits A, B and C of the 2QTS crystal structure include 417, 420 and 418 visible amino acid residues respectively, while the 3HGC crystal structure comprises 406 residues [8], [9]. The corresponding segment of MEC-4 comprises 633 residues for subunit A. Sequence homology based on structural alignment for all residues is 16.4% (104/633) identity and 32% similarity. Most of the residues which have no counterpart in the alignment (Figure 2) are confined in two blocks of sequence (termed NC1 and NC2 herein; depicted as gaps in Figure 2), which correspond to structural elements in the area of the fingertips of the models (Figure 1). Homology between MEC-4 and 2QTS rises to 25.3% identity and 49.5% similarity by excluding the NC1 and NC2 sequence blocks. The 14 cysteines which participate in intramolecular disulphide bridges are at the same positions in both proteins.

Glutamic acid is the most abundant residue through the NC1 and in the first part of the NC2 block. Glutamic acid residues are statistically found on the surface of proteins and are solvent exposed [14]. A homology search [15] with the sequence of NC1 (54 residues) against the protein database (PDB; http://www.wwpdb.org/) revealed 35% identity and 53% similarity with a region of 45 residues in histone H4. The structure of the homologous region comprises a 14-residue loopy region, which adopts different conformations in the two crystallographic studies available (1KX5 chain B and 1EQZ chain D). The remaining 31 residues are part of the first helix-turn-helix motif of histone H4, which is identical in all H4-studies. One striking feature of NC2 (169 residues) is the large number (10) of cysteine residues in the N-terminal part of the sequence. A homology search of the sequence against the protein database revealed limited homology (23% identity, 43% similarity), over a length of 46 residues without insertions or deletions to the Herpes virus entry mediator (PDB IDs: 1JMA and 2AW2). These structures form a two domain rod, are classified as all beta-stranded and posses 8 intramolecular disulphide bonds. If the N-terminal part of NC2 in MEC-4 would adopt a similar conformation, three disulphide bonds would be formed with minimal conformational changes. Similar to the thumb region, these disulphide bonds might contribute to the rigidity of this region. The results of both similarity searches were not considered sufficient enough to model NC1 and NC2. To avoid gaps in the model of MEC-4, short sequences from both sides (N- and C-terminal) of both NC1 and NC2 have been modeled to span the distance between the well aligned residues. The sequence of MEC-4 used in the alignment and the model comprises 432 residues for subunit A.

Four, slightly different subunits were modeled, reflecting the differences in the four subunits. By superimposing these four subunits, one can detect structurally conserved and flexible parts in the models (Figure 1). The palm region, several helices in the finger region and the thumb superimpose extensively. However, there are other parts of the structure, where there are significant differences between the four subunits (loops and turns, mainly in the finger region). In addition, the orientation of the two transmembrane helices, relative to the ‘hand’ differs in the four subunits. Rigidity in the crystal structure of ASIC1 is imposed by the extensive β-sheet and the 7 disulphide bonds. This rigidity is maintained in the degenerin models, assuming that they possess the same structural skeleton built by analogous secondary structure elements.

### Homology-based modeling of UNC-8

A segment of 594 amino acid residues in UNC-8 corresponds to the sequence of 417 residues of 2QTS subunit A. Sequence homology based on structural alignment for these residues is, 17% identity and 30.5% similarity. Low similarity is found in the fingertip region, and particularly in the sequence of the second helix in the structure, which does not align with the sequence of UNC-8. For this reason, 41 residues (ELL…KAN; Figure 2) of 2QTS were left out of the alignment with UNC-8. Thus, one block of 209 residues in UNC-8 remains with no counterpart in 2QTS. A homology search against the PDB database resulted in no significant alignment without extensive gaps for this block of sequence. Limited homology with chain H of mammalian cytochrome BC1 was detected (37% identity and 56% similarity for 37 residues with one gap). The corresponding structural element is mostly helical and was not considered for modeling purposes. Overall homology between UNC-8 and 2QTS rises to 26.2% identity and 47% similarity without the 209 residues. To avoid gaps in the model of UNC-8, short sequences from both sides (5 N- and 6 C-terminal) have been modeled to span the distance between well aligned residues. The sequence of UNC-8 used in the alignment and the model comprises 396 residues for subunit A. In the UNC-8 model, the 7 disulphide bonds are also preserved, with 5 of them located in the thumb region.

### Ion channel mutations

Numerous mutant alleles, which affect ion channel function have been isolated in genetic screens for altered mechanosensory and locomotion phenotypes (Table 1; [5]). The structural impact of these alleles can be examined in the context of the available models. The three *mec-4* alleles which are located in the palm (*u342* (G228S), *u242(ts)* (G234E) and *u209* (S237F), see Table 1) impose severe distortions in the geometry and hydrogen bonding pattern in the beta sheet due to stereochemical hindrance of the large sidechains.

**Table 1:** Ion channel mutants.

Allele	Amino acid substitution	Phenotype	Position in the model	Reference
**MEC-4**				
*u335*	R5Stop	Touch insensitivity	TMI	[27]
*u45(ts)*	G14E	Weak touch insensitivity	TMI	[27]
*u316, e1339*	G79E	Touch insensitivity	Finger	[27]
*u89*	E139K	Touch insensitivity	Finger	[27]
*u342*	G228S	Touch insensitivity	Palm	[27]
*u242(ts)*	G234E	Touch insensitivity	Palm	[27]
*u209*	S237F	Touch insensitivity	Palm	[27]
*e1601, u273, u128, u340*	C290V	Touch insensitivity	Thumb	[27]
*u221*	G293E	Touch insensitivity	Thumb	[27]
*u315*	D294N	Touch insensitivity	Thumb	[27]
*bz149*	R296C	Touch insensitivity	Thumb	[28]
*bz183*	R314K	Suppressor of mec-4(u231) necrosis	Thumb	[28]
*bz195*	S330F	Suppressor of *mec-4(u231)* necrosis	Thumb	[28]
*bz94*	P338L	Suppressor of *mec-4(u231)* necrosis	Thumb	[28]
*bz104*	P350L	Suppressor of *mec-4(u231)* necrosis	–	[28]
*bz112*	W353Stop	Suppressor of *mec-4(u231)* necrosis	–	[28]
*bz150, u72, u441*	Q359Stop	Suppressor of *mec-4(u231)* necrosis	–	[28]
*bz159*	T396I	Suppressor of *mec-4(u231)* necrosis	–	[28]
*u231, u56*	A408V	Neurodegeneration, necrosis	TMII	[20]
*bz173*	G411E	Suppressor of *mec-4(u231)* necrosis	TMII	[28]
*u2*	G412D	Suppressor of *mec-4(u231)* necrosis	TMII	[28]
*bz11*	G412S	Suppressor of *mec-4(u231)* necrosis	TMII	[28]
*bz165*	G415D	Suppressor of *mec-4(u231)* necrosis	TMII	[28]
*u260*	W417Stop	Touch insensitivity	TMII	[27]
*bz139*	C418Y	Suppressor of *mec-4(u231)* necrosis	TMII	[28]
*e1789, u35*	S421F	Touch insensitivity	TMII	[29]
*bz101*	L423F	Suppressor of *mec-4(u231)* necrosis	TMII	[28]
*bz184, u246, u238*	T424I	Touch insensitivity	TMII	[29]
*u29*	E427K	Touch insensitivity	TMII	[29]
*bz8*	L433E	Suppressor of *mec-4(u231)* necrosis	TMII	[28]
**UNC-8**				
*n1193*	E232K	Interrupted locomotion	Thumb	This study
*e49*	A266T	Uncoordinated locomotion	Thumb	[7]
*lb82*	T344I	Abnormal locomotion	–	[7]

The molecular characteristics, the associated mutant phenotypes and the localization of amino-acid substitutions on the model are indicated. TM: Transmembrane.

**Figure 3:**
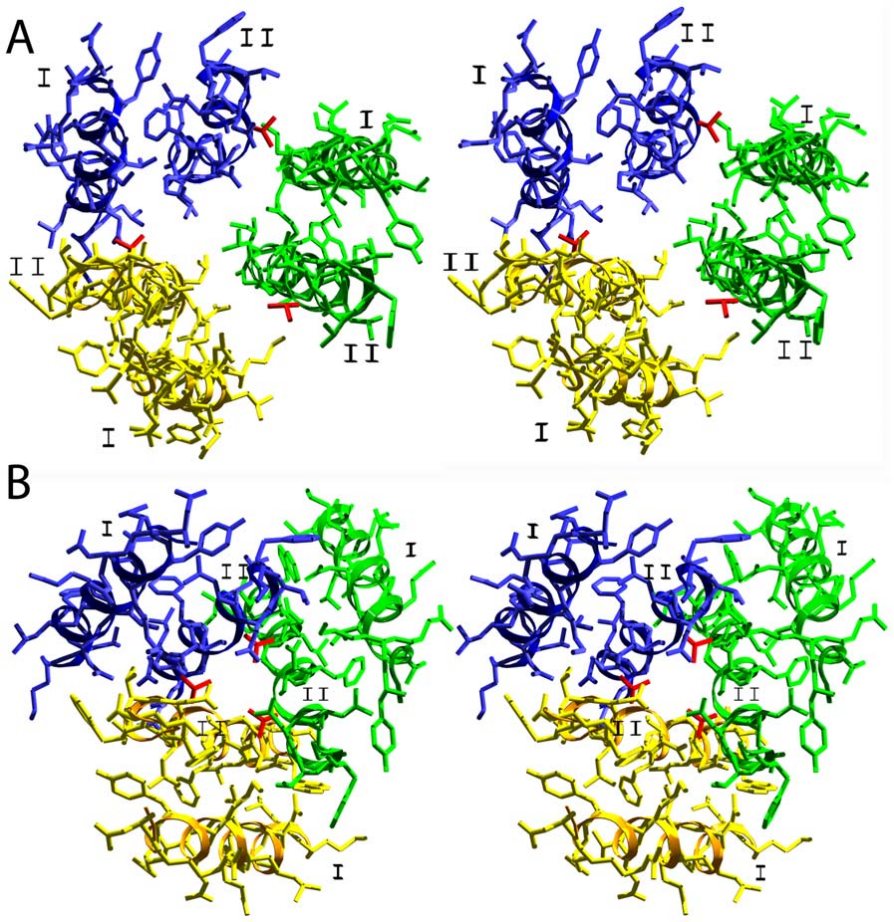
Stereo views of the environment of the dominant, gain-of-function *mec-4* allele *u231* (here A408V). The sidechain of A408 was substituted by Val and is shown in red. I and II denote transmembrane helices I and II. **a**: Yellow, blue and green denote the different subunits of *mec-4* modeled to the three different subunits of the crystal structure 2QTS. **b**: Yellow, blue and green denote the crystallographic equivalent subunits of the trimer derived by application of crystallographic symmetry. The subunit was modeled to the subunit of 3HGC. The same helical segments are shown as in Figure 3a.

Both Val and Ala residues are abundant in TM-helices [16], [17]. The *mec-4* allele *u231* (A408V; also referred to as A713V), encodes a constitutively open ion channel. A408 is located in the second TM-helix. It has been found that the hydrophobic moment [18], [19] is not affected in direction or magnitude by this allele. Substitutions for Gly and Ser at this position have no effect in the function of MEC-4 [2], [20]. Furthermore, Gly and Ser are found in other members of the degenerin family at this position. The model of trimeric MEC-4, modeled on 2QTS shows that Ala408 is facing the first TM-helix of another subunit (Figure 3a). Based on this trimer model, there is no evidence that more bulky sidechains like Val could interfere with the close contact of the helices, as there seems to be enough space to accommodate even bulkier sidechains. The packing moment [21] of the helices is only marginally changed.

The extracellular domain (residues 34–400) of both crystal structures is essentially the same (the RMS deviation for the backbone atoms of all residues, including loops and turns, is 1.65 Å). Both crystal structures differ in the bending angle between the transmembrane helices and the extracellular domain as well as in the arrangement of the transmembrane helices. In 2QTS both transmembrane helices of each subunit pack to form, together with the other subunits, a circle in alternating fashion. The circle built by the three TM-I alone has a marginally larger radius than the circle of the TM-IIs alone. This is reflected in the model of MEC-4 (Figure 3a).

However, a different situation emerges in the case of 3HGC and the trimer model derived from this crystal structure. Here, in the protein derived from the minimal function construct, the TM-II helices build the core and are flanked by TM-Is (Figure 3b). Ala408 is now facing another TM-II and the space available for bulkier sidechains is more restricted. Nevertheless, substituting Ala 408 by Val does not provoke close contacts in this region (Figure 3b). The only close backbone contact (3.5 Å) is with the C = O group of Asn405 of the same chain, which is within the acceptable limit. Other close contacts with the adjacent subunit sidechains Asp409 and Gln413 are avoidable by small conformational changes in these side-chains. Enough space appears to be available, even for bulkier sidechains than Val, without forcing the adjacent subunits to move apart.

The conformational space available for a Val backbone is more restricted in comparison to Ala or Gly. A conformational change at position 408 in the MEC-4 model would let the carbonyl group of residue 408 tip out of the helix axis and thus make this carbonyl group available for coordinating a cation. Such coordination of the corresponding residue (Gly 432) was observed in the structure of ASIC-mfc, when crystals were soaked with Cs^+^ containing solution [8]. Distortions in helix geometry and in the hydrogen bonding patterns in MEC-4 could be compensated by conformational changes of other Glycines in this Gly-rich region. We propose that the A408V allele restricts the position of the carbonyl oxygen at position 408 so as to make coordination of a cation impossible, thus leading to a permanently open ion channel. Other mutant alleles [G411D (*u2*), G411S (*bz173*), G412E (*bz11*) and G415D (*bz165*)] change sidechains 3, 4 and 7 residues downstream of A408V (*u231*). In the proposed models these residues are placed on the same side of the helix as A408, one (G411 and G412) and two turns (G415) towards the cytoplasm. These residues are implicated in gating the channel and certain mutations may alter selectivity [22], [23]. Furthermore, all 4 alleles suppress neurodegeneration caused by A408V. These 4 alleles substitute Gly by residues with carboxyl or hydroxyl group(s) in the sidechain. The backbone conformation of the modeled 408 residues lies strictly in the helical region, as it is in ASIC-mfc. We hypothesize that if Val408 is not allowed to coordinate cations with its carbonyl group due to conformational restrictions of the backbone, the long polar sidechains of the second-site alleles overtake this coordination in the double-mutant channels. The free passage of ions through a permanently open ion channel may be slowed down and/or modulated by these polar residues which protrude from the three subunits inside the ion channel.

**Figure 4:**
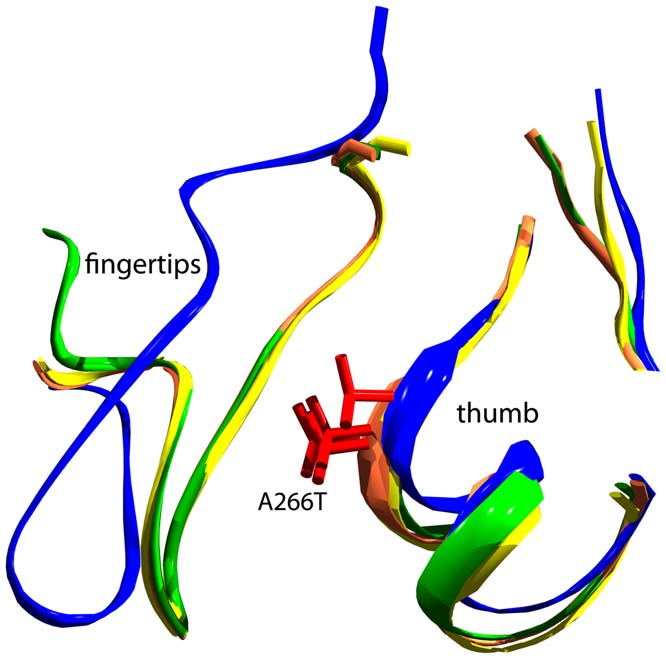
The environment (within 9 Å) of the semi-dominant gain-of-function *unc-8* allele *e49*, here A266T, in the 4 superimposed models of a subunit of UNC-8. For clarity, only ribbons are drawn. Yellow, blue, green and orange denote the different modeled subunits of *unc-8*. The sidechain of A266 was substituted in the picture by T266 and is shown in red.

The *unc-8*(*e49)* allele is semi dominant gain of function allele (here A266T) which results in mild uncoordination of the animals, without neuronal swelling. This residue is situated at the thumb tip and not in a flexible loop, at the beginning of a helix, in the models. The direction of the sidechain points towards the clenched fingertips (Figure 4). Due to limited homology between UNC-8 and 2QTS in this region, it was not modeled except 11 amino acid residues within the two boundaries of this region. Changing Ala266 to the more bulky Thr will sterically influence the neighborhood of the fingertip residues. This substitution might also cause the threonine sidechain to form hydrogen bond(s) with the fingertip and therefore interfere with proper function of the channel.

Homology models of MEC-4 and UNC-8 were obtained based on both available crystallographic structures of the closed chicken acid sensing ion channel of (PDB IDs: 2QTS, 3HGC). The fact that sequences of MEC-4 and 2QTS/3HGC share only 16% identity and consist of 634 and 417/406 residues respectively, coupled with the limited capacity of contemporary modeling software to properly handle insertions and deletions in amino acid sequences render modeling attempts particularly challenging for this class of proteins. We performed knowledge-based modeling, on the assumption that key secondary structural elements, such as α-helices, the long β-sheet and the 7 disulphide bridges are also maintained in the modeled degenerin subunits. The additional stretches of residues, which lie in turns, flexible loops or the larger inserts, form secondary structural elements that were not modeled but may nevertheless be important for function.

Given that MEC-4 and UNC-8 form extensive connections, both with the extracellular matrix and the intracellular environment, structural models should be particularly useful for identifying critical residues involved in structural stability and predicting protein-protein interactions. Functionally important regions of a protein tend to be more highly conserved and thus more accurately modeled. Identification of potential protein partners might reveal shared interactions among the DEG/ENaC family members. In addition, homology modeling can also be used to identify subtle differences among various members of the DEG/ENaC family, which share high sequence similarity. Thus, special structural elements may be linked to functional features. Our models provide a tool for immediate correlation between genotype and phenotype. Modeling of MEC-4 structural alterations induced by the *u231* allele, provides insight about the mechanism of degeneration triggered by this toxic channel derivative. Combined with molecular dynamics simulations, homology models can also generate hypotheses about the kinetics and dynamics of members of DEG/ENaC family, pertaining ion selectivity. These models can also guide mutagenesis experiments, or hypotheses about structure-function relationships. In *C. elegans*, members of the DEG/ENaC family have diverse expression patterns and are present essentially in all tissues. Directed mutagenesis of homology based models will provide a tool for provoking ion imbalance and consequently degeneration in selected tissues. Rational designing of agonists and antagonists will provide tools for temporal control of degeneration. Given that DEG/ENaC variants are potent initiators of necrotic cell death and neurodegeneration, elucidation of the key structural features linked to this capacity will help correlate genotypic and phenotypic mutation data and guide experimental design.

Structural studies are clearly required to test the three-dimensional MEC-4 and UNC-8 models. Such studies are not trivial however, because of inherent difficulties in efficiently expressing degenerins using a heterologous expression system. An additional difficulty arises from the transmembrane nature of these proteins. Our models provide a first approximation to the structure of functionally important domains in these metazoan mechanosensory ion channel subunits, and remains to be tested experimentally.

## Methods

The three subunits of the first trimer of 2QTS (subunits A, B and C) have been superimposed: two subunits to the third in alternating order. The sequences of MEC-4 and UNC-8 were both aligned three times (both to the superimposed subunits ABC, BCA and CAB of 2QTS) in the Swiss-PDB Viewer [24]. For subunit A, B and C in MEC-4, 432, 435 and 433 residues were modeled reflecting the slightly different lengths at the N-and C-terminals of the crystals structure's subunits (417, 420 and 418 residues). The crystal structure of the minimal function channel 3HGC is described by one subunit in the asymmetric unit of a trigonal space group. This structure comprises 406 visible residues to which 421 residues of MEC-4 were aligned in the same way as to the subunits of 2QTS. The sequences of UNC-8 comprise 396, 399 and 397 residues modeled to the 3 superimposed subunits of 2QTS and 385 residues modeled to the subunit of 3HGC. The structural alignments of the 8 subunits (4 MEC-4 and 4 UNC-8) were aligned in alternating order to the superimposed chains of 2QTS and the alignments are submitted separately to the SWISS-MODEL server (http://swissmodel.expasy.org/). Preliminary models with poor geometry were obtained. These models were manually adjusted in order to optimize the geometry of certain regions. The models were energy minimized with the program CNS (*in vacuo*, 1200 cycles of conjugate gradient minimization with hydrogen bond restraints, non-bonded cutoff 13 Å) [25]. The *cis*-prolines observed in 2QTS were also maintained in the models. Two additional *cis*-prolines were introduced in the MEC-4 models A and B and one in C, whereas only one additional *cis*-proline was introduced in the three UNC-8 models. In 3HGC, only one *cis*-peptide bond containing proline and three *cis*-peptides with other residues were determined. Two of these non-proline *cis*-bonds were maintained in the models of MEC-4, and the same additional *cis*-proline residues were introduced as in the subunits modeled to the high resolution structure 2QTS.

In the structural alignment used for modeling of UNC-8 to 3HGC, the only non-proline *cis*-peptide in 3HGC which aligns to the sequence of UNC-8, aligns to a proline in the UNC-8 sequence and was maintained. The C-terminal *cis*-proline was also modeled in UNC-8 like a third *cis*-proline, as in all other models at the same region in the alignment. The geometry of the final models was analyzed using the program PROCHECK [26]. The trimer of MEC-4 modeled to 2QTS was obtained by placing the three final subunits on the trimeric crystal structure (A, B and C) of 2QTS. The trimer modeled to 3HGC was obtained by placing the subunit in the unit cell of 3HGC and applying space group symmetry. Both trimeric models were energy minimized employing the same conditions as in the subunit energy minimizations of the individual subunits. This was considered necessary in order to remove some interchain contacts. The models are accessible at http://elegans.imbb.forth.gr/models/.
